# Overgrowth of Squamocolumnar Junction and Dysregulation of Stem Cell Lineages in the Stomach of Vitamin A-Deficient Mice

**DOI:** 10.3390/nu14163334

**Published:** 2022-08-15

**Authors:** Neethu Vins, Subi Sugathan, Asma Al Menhali, Sherif M. Karam

**Affiliations:** 1 Department of Anatomy, College of Medicine & Health Sciences, United Arab Emirates University, Al Ain P.O. Box 15551, United Arab Emirates; 2 Department of Biology, College of Science, United Arab Emirates University, Al Ain P.O. Box 15551, United Arab Emirates; 3 Zayed Bin Sultan Centre for Health Sciences, United Arab Emirates University, Al Ain P.O. Box 15551, United Arab Emirates

**Keywords:** micronutrients, vitamin A, gastric epithelium, epithelial integrity, squamocolumnar junction, gastric stem cell lineages

## Abstract

Junctional epithelia are common sites for pathological transformations. In mice, the stratified epithelium of the forestomach joins the simple glandular epithelium of the cardia at the limiting ridge. We previously demonstrated the expression of vitamin A receptors in the gastric stem/progenitor cells and their progeny and found that excess retinoic acid enhances cellular dynamics of gastric epithelium. This study examines how deficiency of vitamin A would alter gastric epithelial stem cell lineages. Three-week-old mice of both genders were weaned and fed with a vitamin A deficient (VAD) diet for 4 or 8 months. Sex- and weight-matched littermate mice received a standard (control) diet. To label S-phase cells, all mice received a single intraperitoneal injection of 5-bromo-2-deoxyuridine before being euthanized. Stomach tissues were processed for microscopic examination and protein analysis to investigate stem cell lineages using different stains, lectins, or antibodies. The Student’s *t*-test was used to compare quantified data showing differences between control and VAD groups. Eight-month-vitamin-A deficiency caused enlarged forestomach and overgrowth of the squamocolumnar junction with metaplastic and dysplastic cardiac glands, formation of intramucosal cysts, loss of surface mucosal integrity, increased amount of luminal surface mucus, and upregulation of trefoil factor 1 and H^+^,K^+^-ATPase. These changes were associated with decreased cell proliferation and upregulation of p63. In conclusion, vitamin A is necessary for maintaining gastric epithelial integrity and its deficiency predisposes the mouse stomach to precancerous lesions.

## 1. Introduction

Vitamin A is a multifunctional micronutrient involved in several essential physiological processes such as cell proliferation and differentiation. It is, therefore, required for the integrity of epithelial tissues in many organs including the skin, testes, lungs, eyes, mammary glands, and the intestine [1,2,3,4,5,6]. According to a recent report, vitamin A deficiency is a major health problem in more than half of the world’s countries and it is the most common type of vitamin deficiency worldwide [7].

**Figure 1:**
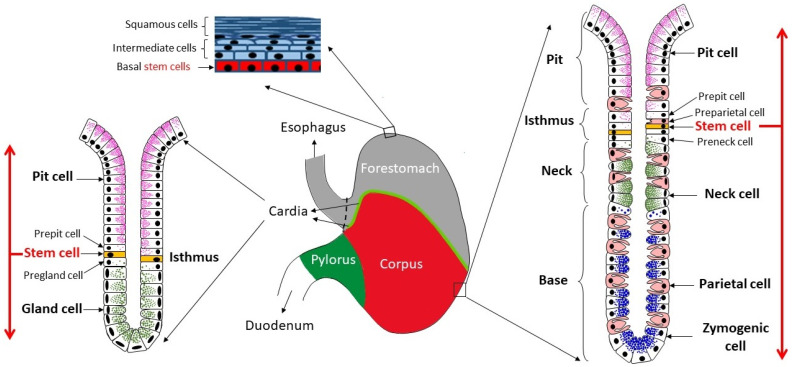
Diagram representing the mouse stomach regions (forestomach, cardia, corpus, and pylorus) with the stratified epithelium of the forestomach and the glandular epithelia of the cardia and the corpus. The progenitor/stem cells in the basal layer of the forestomach and near the middle (isthmus) of the cardiac and corpus glands give rise to squamous cells, mucus-secreting pit, and gland/neck cells, acid-secreting parietal cells, and pepsinogen-secreting zymogenic cells. The red arrows indicate the bi-directional migration pathways of the stem cell progenies.

Very little is known about the role of vitamin A in the stomach. In mice, the stomach is divided into non-glandular forestomach and three glandular regions: cardia, corpus, and pylorus (Figure 1). The forestomach is connected distally with the esophagus and proximally with the cardia at the limiting ridge [8]. While the forestomach is lined by stratified squamous keratinized epithelium, the three glandular regions are lined by a single layer of epithelial cells which invaginates to form innumerable tubular glands responsible for the production of the gastric secretions [8,9]. Each gland is made of a pit, isthmus, neck, and base populated by different types of cells (Figure 1). In the cardia and pylorus, surface mucous (or pit) cells populate the pit and the gland mucous cells dominate in the neck and base. Enteroendocrine cells are scattered throughout the gland. In the corpus, pit, mucous neck, and zymogenic (or chief) cells dominate in the glandular pit, neck, and base, respectively. Parietal and enteroendocrine cells are scattered throughout the gland. In between the pit and neck, the narrow isthmus includes small dividing stem/progenitor cells which give rise to all epithelial cell lineages populating the gastric glands [9,10].

Abnormalities in gastric glands occur during the pathogenesis of some gastric disorders, such as gastritis, ulcers, and tumorigenic transformations. These disorders are also associated with alteration of gastric epithelial cell proliferation and differentiation [11]. Several transcription factors and signaling pathways have been found to control the dynamics of the gastric epithelium, such as Barx1, Sox2, Mist1, trefoil factors (TFF1 and TFF2), Wnt, Notch, hedgehog, and bone morphogenetic proteins [12,13,14].

Little is known about nutritional factors such as vitamins that regulate cell proliferation and differentiation program in the gastric glands. Vitamins are micronutrients that play essential roles in body functions. For example, the active form of vitamin A, retinoic acid, is needed for normal growth and epithelial differentiation of respiratory, epidermal, intestinal, and mammary epithelia [15,16,17]. It has been reported that vitamin A deficiency disrupts epithelial integrity of the conjunctiva, as well as the intestinal and respiratory tracts [18]. In the stomachs of mice and humans, the retinoid receptors have been identified in the gastric epithelium and intensified in the area of stem/progenitor cells. It has been also shown that excess retinoic acid stimulates the dynamics of cellular production and differentiation in the mouse gastric glands [19,20]. In humans, while vitamin A (retinol or retinoic acid) is found to inhibit the proliferation of several gastric cancer cell lines via antioxidant properties, a meta-analysis based on several case reports and cohort studies have confirmed the inverse relationship between dietary intake of vitamin A and gastric cancer development [21]. This association between dietary vitamin A, retinol intake, and blood retinol level, and the risk of gastric cancer implied a possible use for vitamin A in cancer prevention. However, detailed molecular and cellular effects of vitamin A on the human stomach are not well studied. It is also not known yet how deficiency of vitamin A would affect cell lineages in the gastric glands and the proliferation of epithelial stem/progenitor cells. Therefore, the aim of this study was to systematically analyze the proliferation and progeny of gastric epithelial stem/progenitor cells in mice fed with a vitamin A deficient (VAD) diet from the time of weaning up to 4 and 8 months.

## 2. Materials and Methods

### 2.1. Animals

C57BL/6J mice were used in this study and maintained in the animal facility of the UAE University. All mice were kept at room temperature of 22 °C and 55% humidity, on a 12/12 h light/dark cycle and given food and water *ad libitum* throughout the study. The animal procedures were approved by the Animal Ethics Committee of the authors’ institution and are in line with the federal government law and the guidelines of animal welfare.

### 2.2. Experimental Design

Twenty-one-day-old mice were weaned, weighed, and individually separated in cages. Each pair of weight- and sex-matched littermate mice was kept next to each other and labeled as a control and a VAD. The control mice received a standard diet containing vitamin A (Research Diets, New Brunswick, NJ, USA; catalogue number D10012G). The VAD mice received a diet similar to that of control mice but without vitamin A (catalogue number D13111). Mice were maintained for two timepoints of 4 and 8 months. Each timepoint included male and female pairs of control or VAD groups. Each control and VAD group included 6 to 12 mice per timepoint. Therefore, the 4-month mice included 4 pairs of control-VAD males and 5 pairs of control-VAD females. The 8-month mice included 3 pairs of control-VAD males and 6 pairs of control-VAD females. The bodyweight and the intake of food were recorded twice a week. On the last day of each timepoint, all control and VAD mice received a single intraperitoneal injection of 120 mg/kg body weight of 5-bromo-2-deoxyuridine (BrdU; Sigma-Aldrich, St Louis, MO, USA) to label cells in the S-phase of the cell cycle. Two hours later, mice were anesthetized in pairs (control and VAD) and their stomachs were removed, cut along the greater curvature, and processed for microscopic examinations and western blot analysis.

### 2.3. Tissue Processing for Histological, Immunohistochemical, and Lectin Binding Analyses

A longitudinally cut rectangular piece of each stomach that includes part of the forestomach and the glandular region was fixed in Bouin’s solution overnight. Following dehydration, clearing, and impregnation, each pair of control and VAD stomach tissues (obtained from weight- and sex-matched littermate mice) were embedded together in the same paraffin block to minimize individual variations when comparing labeling intensities. Serial 5-µm-thick tissue sections were processed for conventional hematoxylin and eosin staining as well as periodic acid Schiff (PAS) and Alcian blue staining methods. Tissue sections were also processed for different immunoprobing and lectin bindings.

The following were the primary antibodies used for immunohistochemistry: mouse monoclonal anti-BrdU antibody (Medical and Biological Laboratories Co., Nagoya, Japan), anti-pepsinogen C (Abcam, Cambridge, UK), anti-H^+^,K^+^-ATPase β-subunit (Medical and Biological Laboratories Co., Woburn, MA, USA), and anti-p63 antibodies (Dako, Carpinteria, CA, USA). Rabbit polyclonal anti-ghrelin, anti-TFF1, and anti-TFF2 antibodies were kindly provided by Dr. Catherine Tomasetto [13]. Lectins used for this study were fucose-specific *Ulex europaeus* agglutinin 1 (UEA) conjugated to rhodamine (Vector Laboratories Inc., Burlingame, CA, USA) and N-acetyl-D-glucosamine-specific *Griffonia simplicifolia* II (GS) conjugated to fluorescein isothiocyanate (FITC; Thermo-Fisher Scientific, Molecular probes by Life Technologies, Eugene, OR, USA).

The tissue sections labeled with different biomarkers were examined with the Olympus fluorescence microscope connected to digital camera DP70. The JPEG images were taken from three different adjacent areas of the glandular region starting from and including the limiting ridge. The number of labeled cells and/or the percentages of labeled pixels reflecting the labeling intensities were quantified using the Fiji ImageJ software.

### 2.4. Western Blotting

The stomach tissues were homogenized in RIPA buffer (Sigma-Aldrich, St. Louis, MO, USA) to collect the whole cell lysate and proteins were quantified using the Bradford assay. Twenty micrograms of protein were loaded per lane in SDS-PAGE. Separated proteins were then transferred to a nitrocellulose membrane and blocked with 5% skimmed milk at room temperature for 1 h. Primary antibodies specific for p63 and H^+^,K^+^-ATPase β-subunit in blocking solution were added and incubated overnight at 4 °C with gentle agitation. Probed membranes were incubated with the appropriate secondary antibodies conjugated with horseradish peroxidase for 1 h at room temperature. Finally, the protein bands were detected using SuperSignal West chemiluminescence substrate (Thermo Scientific, Rockford, IL, USA) and the documentation system Typhoon FLA 9500 (GE Healthcare Bio-sciences AB, Uppsala, Sweden).

### 2.5. Statistical Analysis

To compare the differences between control groups and VAD groups, statistics were performed using the unpaired Student’s *t*-test. The results were presented as mean ± standard errors (SE). P values less than 0.05 were considered statistically significant.

## 3. Results

### 3.1. Differential Decrease in Body Weight and Food Consumption in VAD Mice

**Figure 2:**
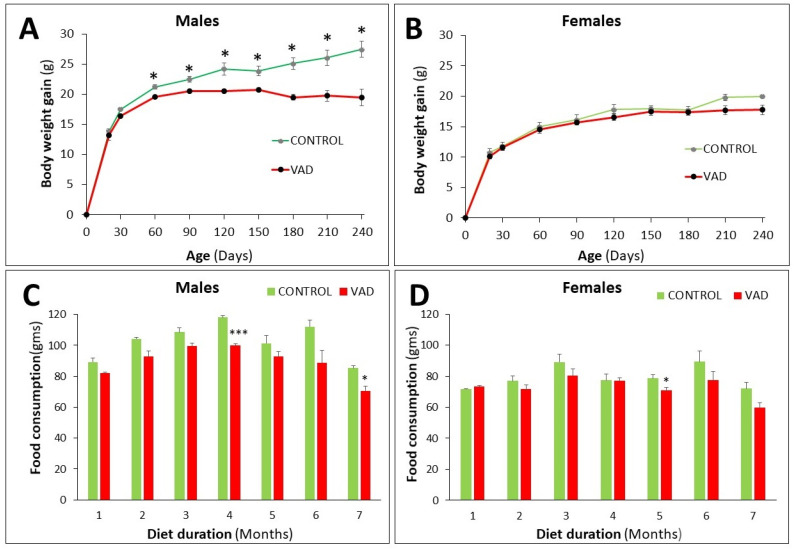
Weight gain (**A**,**B**) and food consumption (**C**,**D**) in control and VAD 8-month mice. Monthly changes are recorded separately in male (*n* = 6) and female (*n* = 12) mice. Each timepoint represents the mean ± SE. The asterisks indicate the significance of the difference between control and VAD, * *p* < 0.05, *** *p* < 0.001.

During the course of this study, mice were put on standard (control) or VAD diets for 4 or 8 months. In the control mice, males gained more body weight than females (Figure 2A,B). In VAD mice, males showed a significant decrease in body weight gain when compared with their control littermates from 2 months (*p* = 0.018) onwards (Figure 2A). However, female VAD mice, did not show any significant change in their body weight gain as compared to their control littermates (Figure 2B).

Changes in food consumption in control mice showed a trend similar to that of their body weight gain. Males consumed more food than females (Figure 2C,D). In VAD mice, food consumption was reduced in males when compared to their control littermates and the difference was statistically significant only at 4 and 7 months, *p* = 0.0006 and 0.025, respectively (Figure 2C). In female VAD mice, the reduction in food consumption was only significant at 5 months, *p* = 0.045 (Figure 2D).

### 3.2. Anatomical and Histological Alterations of VAD Stomachs

Gross morphological examination of the stomachs of 4-month-VAD mice showed no difference when compared with their control littermates. However, microscopic examination of the stomach tissue sections of the 4-month VAD mice stained with H&E or PAS showed a few mild glandular dilatations in the cardiac region near the limiting ridge when compared to their control littermates (Figure S1A).

**Figure 3:**
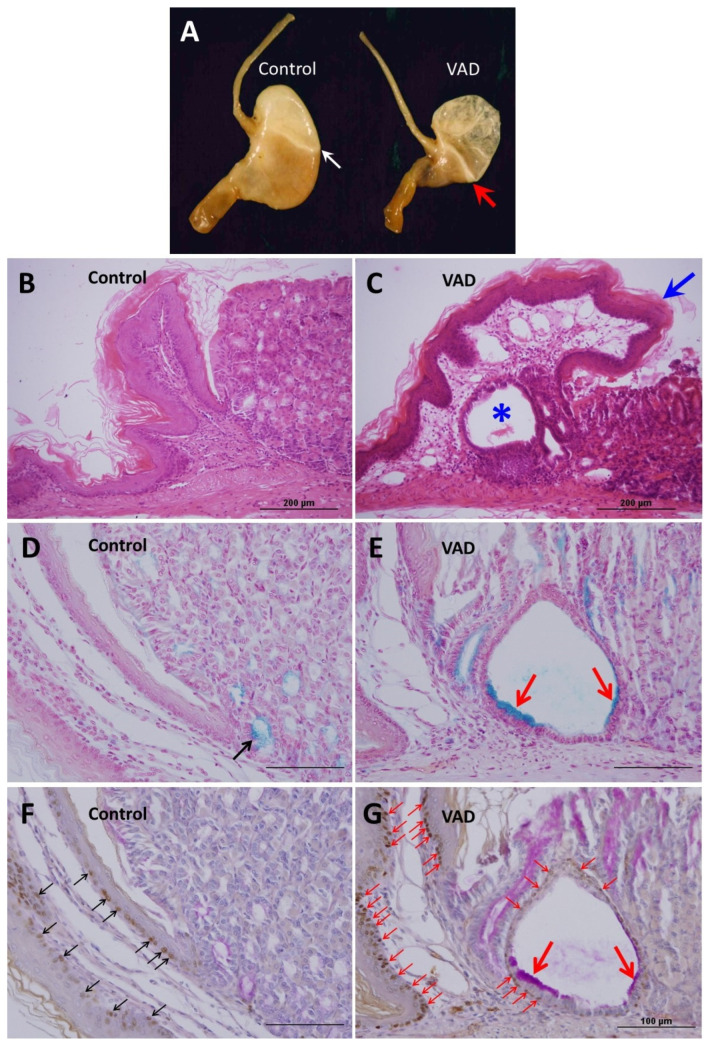
Characterization of the stomachs in 8-month control (**A**,**B**,**D**,**F**) and VAD (**A**,**C**,**E**,**G**) mice. (**A**) Enlarged forestomach of VAD mouse with prominent limiting ridge (white vs. red arrows) at the border with glandular region. (**B**,**C**) Tissue sections of control (**B**) and VAD (**C**) stomachs stained with H&E showing the protruding limiting ridge (blue arrow) folding over the luminal surface of the glandular region (arrow). Note the cystic structure in the VAD tissue (asterisk). (**D**,**E**) Alcian blue staining in control (**D**) and VAD (**E**) tissue shows a few bluish mucous cells in the bottom of the cardiac glands. The mucosal cyst of VAD (**E**) tissue shows Alcian blue positive mucus-rich luminal cells. (**F**,**G**) Immunolocalization of p63 showing positive staining in the basal layer of the stratified squamous epithelium of forestomach in control (**F**, multiple black arrows) and in VAD (**G**) tissue, demarcating the epithelial basal layer of both forestomach and the mucosal cyst (multiple small red arrows). Counterstaining with PAS labels the luminal mucous cells of the cyst (big arrows). Scale bar = 200 µm (**B**,**C**) and 100 µm (**D**–**G**).

By 8 months, the VAD mice developed a strikingly enlarged forestomach at the expense of the glandular region (Figure 3A). In addition, the limiting ridge between the forestomach and the cardia was more prominent in VAD mice than in their control littermates. These observations were more evident in male than female VAD mice (Figure S1A). Microscopically, the 8-month VAD mice were characterized by overgrowth and folding of the limiting ridge over the glandular cardia and corpus (Figure 3B,C). This overgrowth was associated with the enlargement of dilated glandular structures at the limiting ridge between the forestomach and the cardia (Figure 3C) where the stratified squamocolumnar epithelium formed the junction. This dilatation developed a sac-like appearance with an accumulation of luminal secretory material and occasional cellular debris. Therefore, these dilatations appeared similar to intramucosal glandular cysts. At a 4-month diet duration, about 75% and 43% of the male and female VAD mice, respectively, developed mild dilated cardiac glands (Figure S1A–D). By 8 months, all VAD male mice developed cysts whereas only 50% of the females showed this abnormality (Figure S1A,B).

To characterize the cells lining the cystic dilatations, the histochemical Alcian blue and PAS staining methods were initially used. In VAD mice, in addition to the few cells at the base of the cardiac glands, there was an intense Alcian blue staining in some luminal cells lining the cysts compared to fewer stained cells in controls (Figure 3D,E). The Alcian blue-positive cells lining the cysts of the VAD stomach were also stained positive for PAS (Figure 3F,G). Microscopic examination at high magnification, showed that the wall of the cysts was not only lined by columnar mucous cells, but also included an additional basal layer. These small basal cells were immunolabeled with antibodies specific for p63, a transcription factor known to be expressed in the basal cells of stratified epithelia. In the control stomach, the expression of p63 was only observed in some basal cells of the stratified epithelia of the forestomach, but not in the glandular stomach (Figure 3F,G). Figure 3F and Figure S1E,F demonstrate the p63 positive basal cells in the wall of the mucosal cysts of VAD mice. Therefore, the mucosal cysts are lined by stratified epithelium made of two layers, p63-positive basal layer and an Alcian blue-positive and PAS-positive luminal mucous cell layer (Figure 3F,G and Figure S1E,F). Some of the luminal cells lining the mucosal cysts appeared to have less mucus or even no visible mucus, which might indicate partially differentiated mucous cells or progenitor cells.

**Figure 4:**
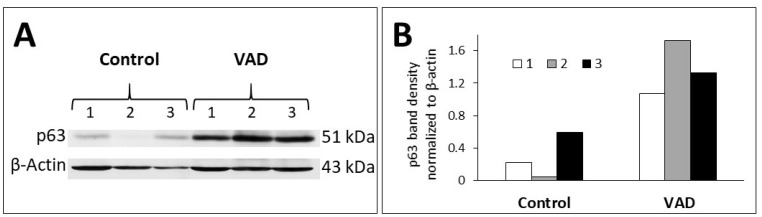
**(A)** Western blot analysis showing p63 protein bands in gastric tissues obtained from three pairs of male 8-month-VAD mice and their control littermates. Beta actin was used as an internal standard. (**B**) Quantification using ImageJ densitometry and β-actin as internal control reveals p63 upregulation in three VAD stomachs.

To confirm and quantify p63 expression, western blot analysis was performed for tissues obtained from the area of the squamocolumnar junction that includes the limiting ridge. As shown in Figure 4A,B, protein analysis using anti-p63 antibodies shows weak expression in control mice and upregulation in VAD mice.

### 3.3. Loss of Mucosal Integrity of VAD Stomach

**Figure 5:**
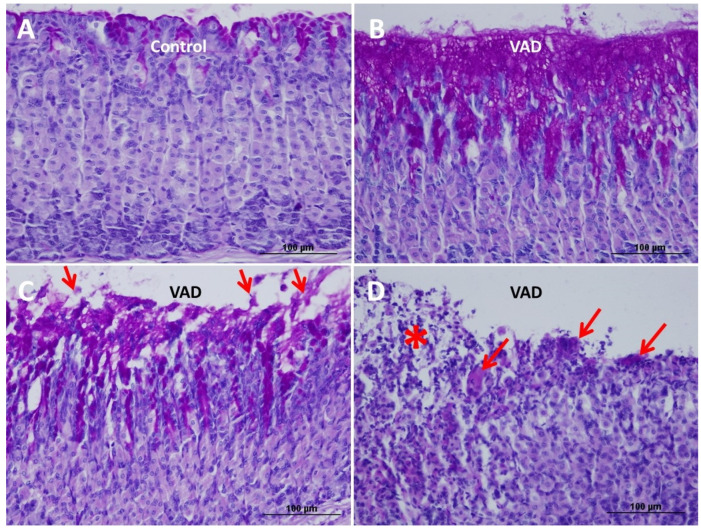
Gastric mucosal tissue sections obtained from 8-month control (**A**) and VAD (**B**–**D**) mice stained with PAS and hematoxylin. (**A**) Control tissue with PAS-positive mucous cells which appear purple at the luminal surface and along the short pits. (**B**) VAD mucosal tissue with massive amount of PAS-stained mucus seen at the luminal surface and along the pits. (**C**) VAD gastric mucosa with sloughing of the PAS-stained mucus and cells (arrows) at the luminal surface. (**D**) VAD gastric mucosa showing loss of surface integrity with sloughing cells (asterisk) and mucinous clumps (arrows) with leucocytic infiltration in the mucosa. Scale bars = 100 µm (**A**–**D**).

Control and VAD gastric tissues of the 4-month mice showed intact, PAS-stained surface epithelium continuous with the gastric glands. However, by 8 months, VAD mice developed multiple areas of discontinuity in the surface epithelium covered with a thick PAS-stained layer of mucus (Figure 5A,B). This layer was frequently seen undergoing sloughing and separation from the luminal surface (Figure 5C). Therefore, in the glandular region of the VAD stomach, the epithelial integrity was lost in several areas with glandular disorganization (Figure 5D). These areas were more frequent in male than female mice. The loss of mucosal integrity was occasionally associated with the infiltration of lymphoid cells (Figure 5D).

### 3.4. Inhibition of Cell Proliferation in VAD Gastric Epithelium

**Figure 6:**
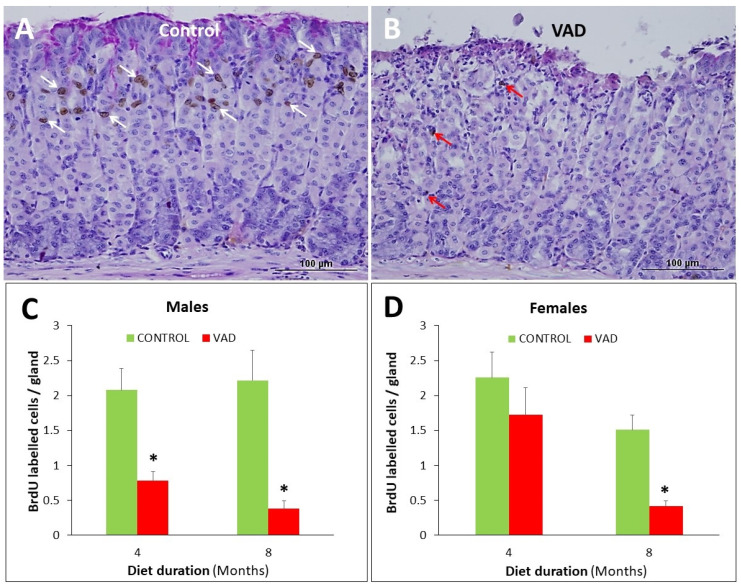
Cell proliferation studies. Gastric mucosal tissue sections from 8-month control (**A**) and VAD (**B**) mice injected with BrdU and probed with anti-BrdU antibodies and counterstained with PAS and hematoxylin. In the control tissue, brownish nuclei of BrdU-labeled cells (white arrows) are seen in the isthmus near the luminal surface. In VAD tissue, only a few BrdU-labeled cells are seen scattered in the mucosa (red arrows). Counts of BrdU-labeled cells in control (**C**) and VAD (**D**) glandular mucosae of male and female mice expressed as average cells per gland ± SE. Scale bars = 100 µm; * *p* < 0.05.

BrdU-labelling was used to identify proliferating cells in the gastric epithelium of control and VAD mice (Figure 6A–D and Figure S2A,B). In the corpus region, the 4-month male mice showed a decrease in cell proliferation from 2.08 BrdU-labelled cells per gland in control to 0.78 in VAD mice, *p* = 0.037 (Figure 6C). However, in female mice, the difference between control and VAD (2.26 vs. 1.73 BrdU positive cells per gland, respectively) was not significant (Figure 6D). By 8 months, cell proliferation was significantly reduced in both males and females (Figure 6A,B). In male mice, the control had 2.22 BrdU cells/gland which was reduced to 0.38 cells/gland in VAD mice, *p* = 0.026. In female mice, the control and VAD groups had 1.51 and 0.38 BrdU-labelled cells per gland, respectively (*p* = 0.016).

### 3.5. Dysregulation of Mucus- and TFF-Secreting Pit and Neck Cells in VAD Stomachs

**Figure 7:**
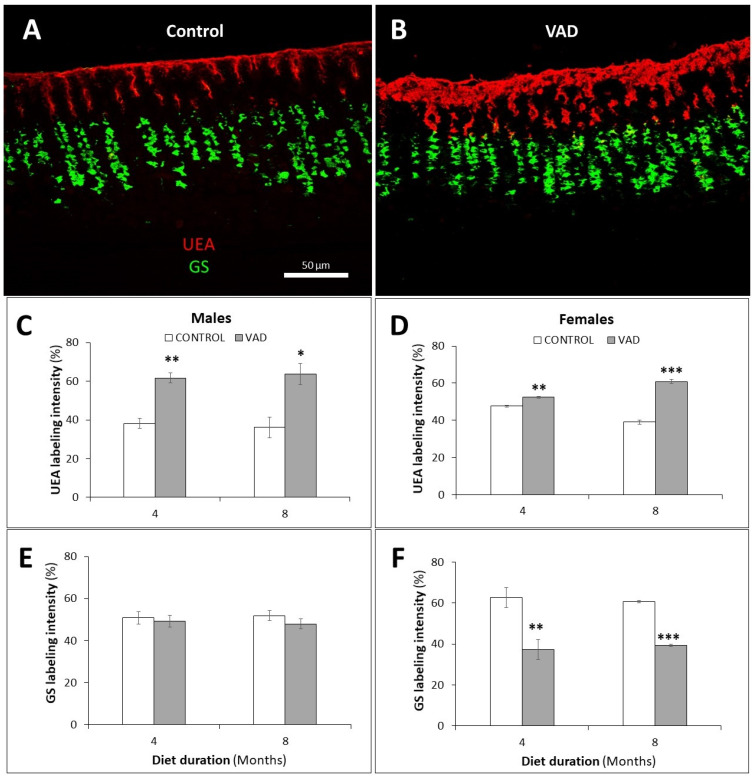
Lectin labeling of mucus-secreting pit and neck cells in the gastric glands of control (**A**) and VAD (**B**) male mice. Rhodamine-conjugated UEA (red) and FITC-conjugated GS (green) lectins bind to pit and neck cells, respectively. The labeling intensities of UEA (**C**,**D**) and GS (**E**,**F**) in control and VAD glandular mucosae of male and female 4- and 8-month mice are presented as mean ± SE. Scale bars in A and B = 50 μm. * *p* < 0.05, ** *p* < 0.01, and *** *p* < 0.001.

The mucus-secreting pit and neck cells were examined by lectin histochemistry. The fucose-specific UEA lectin was used for the detection of pit cells and the *N*-acetylglucosamine-specific GS lectin, for neck cells. The 4- and 8-month VAD male mice showed a significant increase (*p* = 0.0014 and 0.042, respectively) in the UEA staining intensity of mucus in pit cells (Figure 7A–C and Figure S2C,D), with no significant change in GS labeling (Figure 7A,B,E). In the female group, 4- and 8-month VAD mice showed an increased amount of UEA labeling of mucus in pit cells, *p* = 0.003 and 0.0003, respectively (Figure 7D). This was associated with a significant reduction in the GS-labeled mucus of VAD neck cells at 4 and 8 months, *p* = 0.008 and 0.000001, respectively (Figure 7F).

**Figure 8:**
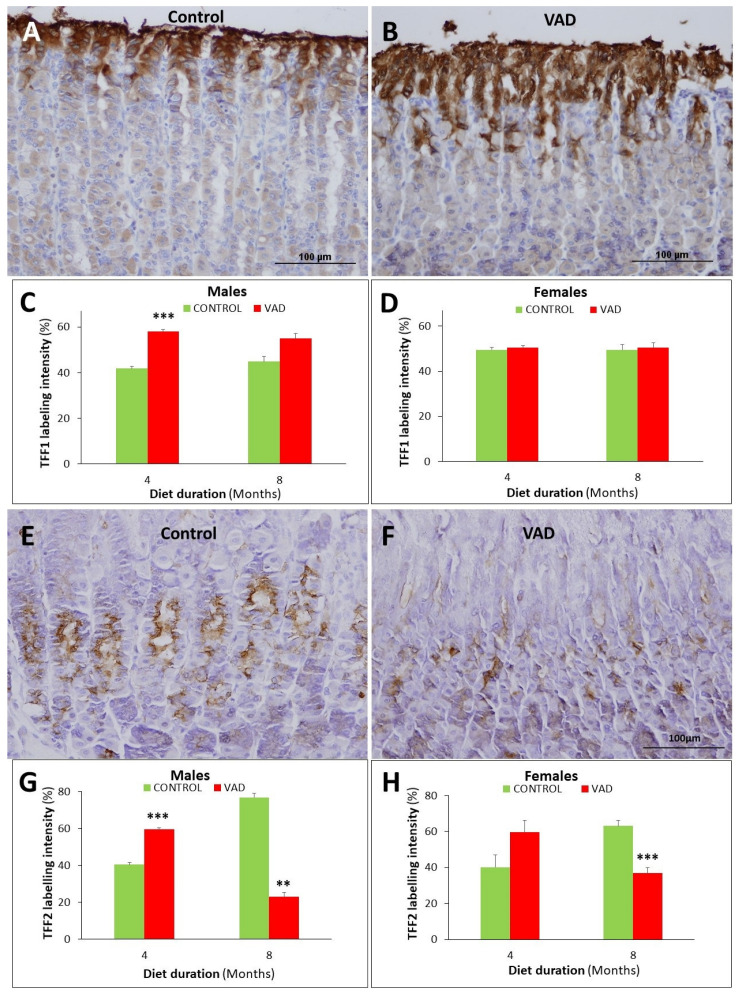
Immunolocalization and quantification of TFF1 (**A**–**D**) and TFF2 (**E**–**H**) in the gastric glands of control and VAD mice. Images are taken from 8-month control (**A**,**E**) and VAD (**B**,**F**) male mice. TFFs immunolabeled cells appear brownish. Cells expressing TFF1 are seen at the luminal surface and the gastric pits (**A**) and are more abundant in VAD tissues than in control (**B**). TFF2 is located in the cells of the neck segment of the gastric glands (**E**) and quantification demonstrates a decreased labeling intensity in VAD tissues (**F**,**H**). Data were presented as mean ± SE. Scale bars = 100 μm. ** *p* < 0.01, and *** *p* < 0.001.

Since pit and neck mucous cells also produce TFFs which are associated with mucus and involved in mucosal protection, tissue sections of control and VAD stomachs were probed with antibodies specific for TFF1 and TFF2. The 4- and 8-month VAD male mice showed an increase in TFF1 labeling intensity (Figure 8A–D and Figure S3A,B). However, the differences were only significant in the 4-month group, *p* = 0.00005, not in the 8-month, *p* = 0.060. In female mice, no significant change was detected in the TFF1 labeling of control and VAD mice at both 4 and 8 months (Figure 8D).

Regarding TFF2, gastric mucosal tissue sections of the 4- and 8-month control and VAD mice showed its localization in the neck segments of the glands (Figure 8E,F and Figure S3C,D). Quantification of the percentage of immunolabeled pixels in images of TFF2 probed sections revealed its significant increase (*p* = 0.00008) in the 4-month VAD male tissues (Figure 8G). However, at eight months, there was a significant reduction (*p* = 0.0061) in the immunolabeling of TFF2 by more than 3-fold (Figure 8G). A similar trend was observed in female VAD mice; TFF2 labelling insignificantly increased at 4-month (*p* = 0.10) and then, significantly reduced at 8-month (*p* = 0.0002) when compared to control littermate mice (Figure 8H).

### 3.6. Increased Proton Pump in Parietal Cells of VAD Stomachs

**Figure 9:**
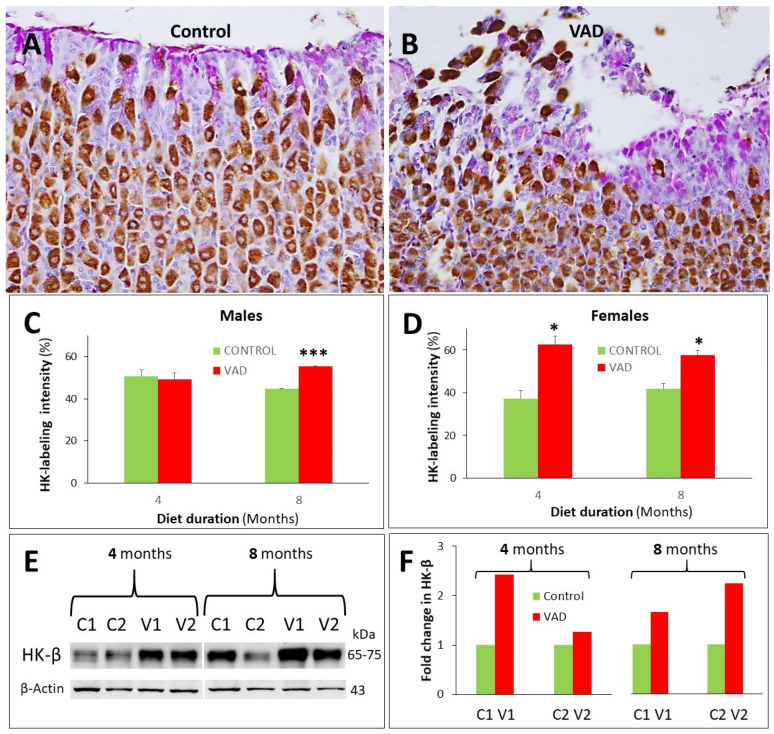
Immuno-analysis of H^+^, K^+^-ATPase in the gastric mucosae of control and VAD mice. Probing of 8-month control (**A**) and VAD (**B**) male stomach tissues shows brownish parietal cells expressing H^+^,K^+^-ATPase and scattered along the gastric glands (**A**,**B**). Quantification demonstrates increased parietal cell labeling intensity in VAD tissues (**C**,**D**). Western blotting using gastric mucosal homogenates from two pairs of control and VAD 4- and 8-month mice shows upregulation of H^+^,K^+^-ATPase (**E**,**F**). Representative individual ImageJ data are normalized using β-actin and presented as fold change relative to the control. Scale bars = 100 μm. * *p* < 0.05, and *** *p* < 0.001.

Immunolabeling using antibodies specific for the H^+^,K^+^-ATPase or the proton pump was used to evaluate the effect of vitamin A deficiency on the acid-secreting parietal cells. While the 4-month male mice showed no significant difference between control and VAD mice, the staining intensity of parietal cells in VAD mice at 8 months was significantly increased, *p* = 0.000008 (Figure 9A–C). In female VAD mice at both 4 and 8 months, there was a significant increase in H^+^,K^+^-ATPase labelling intensity, *p* = 0.017 and 0.016, respectively (Figure 9D and Figure S4A,B). These results were confirmed by western blot analysis (Figure 9E,F).

### 3.7. Ghrelin-Secreting Enteroendocrine Cells Are Reduced in Female VAD Gastric Glands

**Figure 10:**
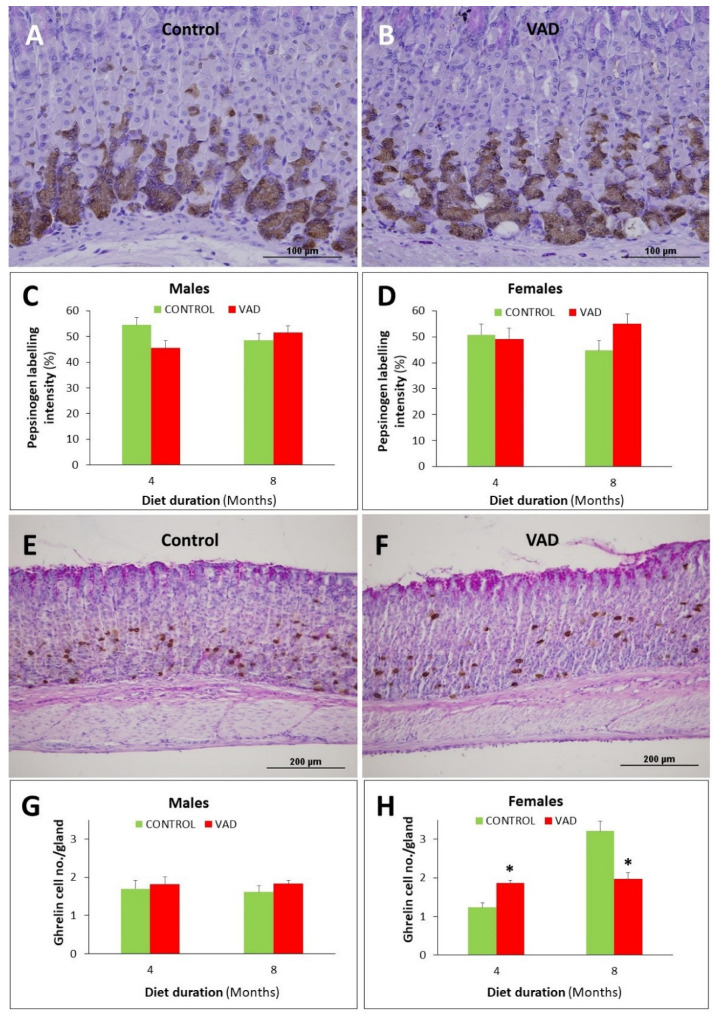
Immunolocalization and analysis of pepsinogen (**A**–**D**) and ghrelin (**E**–**H**) secreting cells in the gastric mucosae of control and VAD mice. Images are taken from 8-month control and VAD male mice. Zymogenic cells expressing pepsinogen appear brownish in the base segment of the gastric glands (**A**,**B**). Quantification demonstrates no significant difference in the labeling intensity of control (**C**) versus VAD (**D**) tissues. Ghrelin-secreting cells are scattered in the gastric mucosae of control (**E**) and VAD (**F**) mice and their numbers are increased in female 4-month VAD mice (**H**) and reduced by 8 months. Scale bars = 100 (**A**,**B**) and 200 (**E**,**F**) μm. * *p* < 0.05.

Immunolocalization of pepsinogen in control and VAD mice did not reveal a significant difference in zymogenic cells of 4- and 8-month mice of either male or female mice (Figure 10A–D and Figure S4C,D). However, quantification of the number of ghrelin-secreting enteroendocrine cells in 4-month VAD female mice revealed a 1.5-fold increase when compared to control, *p* = 0.03 (Figure 10E–H and Figure S4E,F). By 8 months, the situation was reversed and the numbers of ghrelin-positive cells were significantly reduced in VAD female mice (Figure 10H), *p* = 0.012.

## 4. Discussion

The present study demonstrates the importance of vitamin A in maintaining normal structural and biological features of the mouse stomach. In addition to the reported consequences known to occur in several organs due to deficiency of vitamin A [1,2,3,4,5,6], the stomach and gastric epithelial stem cell lineages are seriously affected. It was previously reported that VAD mice and rats develop a reduction in food consumption and body weight [22,23]. The VAD mice of the current study developed a gradual decrease in both body weight gain and food intake which are more evident in males than in females. Surprisingly, these findings are not associated with a change in ghrelin-secreting cell number per gland. But this does not exclude the possible reduction in the total number of ghrelin secreting cells per stomach and the level of circulating ghrelin which likely occurred due to a reduction in the size of the glandular region of the stomach.

A striking feature observed in the 8-month-VAD mice is the overgrowth of the forestomach at the expense of the glandular region. The squamocolumnar junction or the transition zone is a common area for the development of metaplastic and malignant changes [24,25]. In this zone, most of our 4-month-VAD mice have acquired cardiac glands with a dilated lumen which, by 8 months, formed large mucosal cysts which accumulated secretory material and cellular debris in the lumen. Histochemical analysis has demonstrated that these cysts are lined by a mix of small, poorly differentiated cells adjacent to highly differentiated mucous cells stained with both PAS and Alcian blue.

The positive Alcian blue staining in the lining of cystic dilatations indicates the accumulation of sulphated mucopolysaccharides and might represent an early metaplastic change. A faint Alcian blue positive staining was previously observed in the cardiac glands adjacent to the junctional epithelium of control mice [26]. In the normal mouse stomach, these gland mucous cells initially secrete a small amount of sulphated mucin and as they migrate to the gland bottom, they gradually lose this ability and secrete predominantly neutral mucopolysaccharides. Alcian blue staining in the cystic dilatation indicates a block in the differentiation of gland mucous cells and accumulation of increasing amounts of sulphated mucins [27]. This deviation in the normal process of cardiac gland cell differentiation represents an early metaplasia that might precede intestinal metaplasia with the eventual formation of goblet cells. This fascinating cell biological process of transdifferentiation requires further studies.

In addition to the superficial mucous cell layer, the lining epithelium of the mucosal cysts includes a flattened basal cell layer. It was immunolabeled for p63 which is known to be expressed in basal cells of stratified epithelia in different organs including the forestomach. Therefore, the cystic dilatations might have been generated by stem cells with dual features combining those of stem cells that give rise to stratified squamous epithelium of the forestomach and those that give rise to mucus-secreting columnar epithelium of cardiac glands. Previous studies suggested that such a dual feature of an epithelium is considered as a precursor for the development of intestinal metaplasia or Barrett’s esophagus [28]. In another mouse model, the multi-layered epithelium at the squamocolumnar junction of the stomach was also positive for p63 immunolabeling. It was, therefore, classified as columnar metaplasia but not sufficient to develop the intestinal metaplasia [29]. It will be challenging and interesting to investigate the molecular mechanisms and signaling molecules involved and to pinpoint the cellular origin of these VAD-derived mucosal cysts.

This study demonstrates the loss of mucosal integrity in the 8-month VAD stomachs. Intact epithelial lining of the stomach is necessary for protection as the first line of defense against pathogenic organisms and luminal insults [30]. Loss of epithelial integrity was evident in 8-month VAD mice, especially in males and was associated with some inflammatory reaction as demonstrated by mucosal infiltration with leukocytes. VAD was previously shown to enhance the damage of the intestinal mucosa in rats [31]. Hamsters were more sensitive than other rodents and within 7-month VAD developed gastric ulcers which in some cases were associated with bleeding due to perforation of the stomach wall [32]. In our study, mice did not develop ulcers, but the loss of superficial mucosal integrity was associated with streaks of blood on the luminal surface of the stomach. Loss of integrity was also associated with a decrease in epithelial cell proliferation. These findings correlate with our previous study when excess retinoic acid in mice was found to enhance the proliferation of the gastric epithelial stem/progenitor cells [20]. Therefore, the finding that gastric epithelial cell proliferation is inhibited in VAD mice is not unexpected.

In this study, UEA and GS lectins were used as markers for mucinous glycoproteins rich in fucose and N-acetylglucosamine of pit cells and neck cells, respectively. In VAD mice, the amount of mucus at the luminal surface was significantly increased in both male and female mice at 4- and 8-month VAD. A possible interpretation for an increased amount of mucus in VAD mice is that reduction of cell proliferation might be associated with decreased dynamics of surface mucous cells to make them migrate slower and survive longer as a defense against loss of mucosal integrity. This process might lead to the accumulation of mucous granules in cells along the pit and at the luminal surface. When the cells are no longer able to maintain such large amounts of mucous granules, suddenly the cell membranes rupture with a massive release of mucus at the luminal surface. The increased amount of mucus in pit/surface cells was associated with a reduction in the amount of mucus in neck cells of 8-month VAD mice as indicated by GS lectin binding. It will be interesting to investigate the dynamics of mucus secretion and to determine the mechanisms of its dysregulation in VAD mice.

TFFs are mucin-associated peptides that play a major role in the stability of the mucosal barrier and the repair of damaged mucosa [33]. During an acute gastrointestinal mucosal injury, TFF peptides contribute to the stimulation of cell migration for healing of the damaged area. Chronic inflammation also leads to the induction of TFF expression for preventing the further progression of the disease [34]. TFF1 is predominantly expressed in the pit cells. Immunolabeling of TFF1 revealed its upregulation in both 4- and 8-month VAD male mice. This is not surprising since TFF1 is co-secreted and directly packed with mucus in the secretory granules of surface/pit cells [13]. In female VAD mice, the expression of TFF1 was not significantly different from the control at 4 and 8 months. This might be explained by the fact that TFF1 is an estrogen-regulated gene [34,35].

TFF2 is a product of mucous neck cells in the middle of the gastric glands and is colocalized with mucin in the same secretory granules leading to non-covalent interaction with mucin-6 [33,36]. The decrease in GS lectin binding and TFF2 expression in VAD male and female mice at 8 months might correlate with the reduction in cell proliferation and increased proton pump in the gastric mucosa. Therefore, the inhibition of cell proliferation and stimulation of proton pump production is not only due to a lack of TFF2 as previously demonstrated [37], but also due to its downregulation as shown here in VAD mice.

This study demonstrates that VAD mice develop parietal cells with an increased amount of H^+^,K^+^-ATPase. This observation could be due to upregulation of synthesis and/or increased lifespan of H^+^,K^+^-ATPase in parietal cells. In a previous study on isolated canine parietal cells, bone morphogenetic protein-4 was found to upregulate of H^+^,K^+^-ATPase expression and was linked to phosphorylation of Smad1 transcription factor [38]. It will be interesting to determine whether upregulation of H^+^,K^+^-ATPase in VAD mice follows a similar BMP4-Smad1 pathway or is just due to downregulation of TFF2 or due to another mechanism involving estrogen receptors which are expressed in parietal cells [39].

It is known that estrogen receptors are regulated by vitamin A [40]. However, still there is a dispute in the literature regarding the relationship between ghrelin-secreting cells and estrogen. While it has been indicated that ghrelin-secreting cells may be estrogen-dependent [41], some other reports showed that neither estrogen nor testosterone affects ghrelin-secreting cells [42]. In the current study, ghrelin-secreting enteroendocrine cells were significantly affected in female VAD mice. Their number was initially increased in 4-month-VAD mice and then significantly reduced by 8 months. With these conflicting data in the literature and the ghrelin findings of the present study, there is a need for further investigations to clarify this issue.

## 5. Conclusions

**Figure 11:**
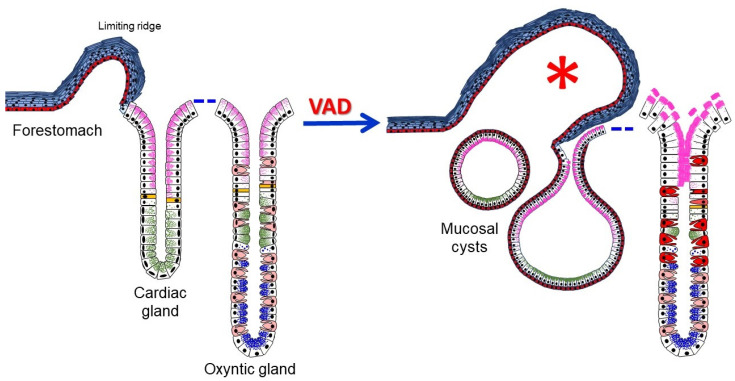
Schematic diagram of control and VAD squamocolumnar junctional epithelium. In the control stomach, the stratified squamous epithelium forms a small limiting ridge that merges with the glandular epithelium of the cardia. The cardiac gland includes a few stem cells (yellow) that give rise to two mucous cell lineages (pink and green granules). The control oxyntic gland also includes dividing stem cells (yellow) that give rise not only to mucous cell lineages (with pink and green granules), but also to parietal cells (nongranular pink cytoplasm). Mucous neck cells contribute mostly to form zymogenic cells (with blue granules) at the bottom. The 8-month VAD tissue develops protrusion with overgrowth of limiting ridge (asterisk), mucosal cysts lined by 2 layers of cells and express P63 at the transition zone, loss of integrity of surface epithelium, reduction in cell proliferation, and increased H^+^,K^+^-ATPase expression in the scattered parietal cells (dark red).

This study demonstrates that VAD mice develop metaplastic and dysplastic transformations at the squamocolumnar junctional epithelium of the limiting ridge with inhibition of stem/progenitor cell proliferation and loss of epithelial integrity with dysregulations of various markers of cell lineages involved in the production of protective and aggressive factors in the gastric glands (Figure 11). These findings highlight the importance of vitamin A in maintaining the integrity of the gastric epithelium with balanced cellular proliferation, differentiation, and death. Therefore, if these findings are found applicable to humans, measurements of vitamin A levels in the clinical setting and its administration might be useful as a preventive measure against some gastric carcinogenesis.

## Data Availability

Raw data generated during the study are available upon reasonable request.

## Supplementary Materials

The following supporting information can be downloaded at: https://www.mdpi.com/article/10.3390/nu14163334/s1, Figure S1: Percentages and histological features of cyctic dilatations in 4- and 8-month mice; Figure S2: BrdU and lectin labeling of 4-month control and VAD mice; Figure S3: TFF1 and TFF2 labeling in 4-month control and VAD mice; Figure S4: Parietal, zymogenic and enteroendocrine cell labeling in 4-month control and VAD mice.
