# Supplementary material for: Overgrowth of Squamocolumnar Junction and Dysregulation of Stem Cell Lineages in the Stomach of Vitamin A-Deficient Mice
Source: Nutrients. 2022 Aug 15;14(16):3334. doi: 10.3390/nu14163334 (PMC9412427; doi:10.3390/nu14163334)
Supplement: Supplementary file 1 [file nutrients-14-03334-s001.zip › nutrients-1848227-supplementary.pdf]

Article

Supplementary Material

Neethu Vins, Subi Sugathan, Asma Al Menhali and Sherif M. Karam:

"Overgrowth of Squamocolumnar Junction and Dysregulation of Stem Cell Lineages in the Stomach of Vitamin A-Deficient Mice"

Figure S1

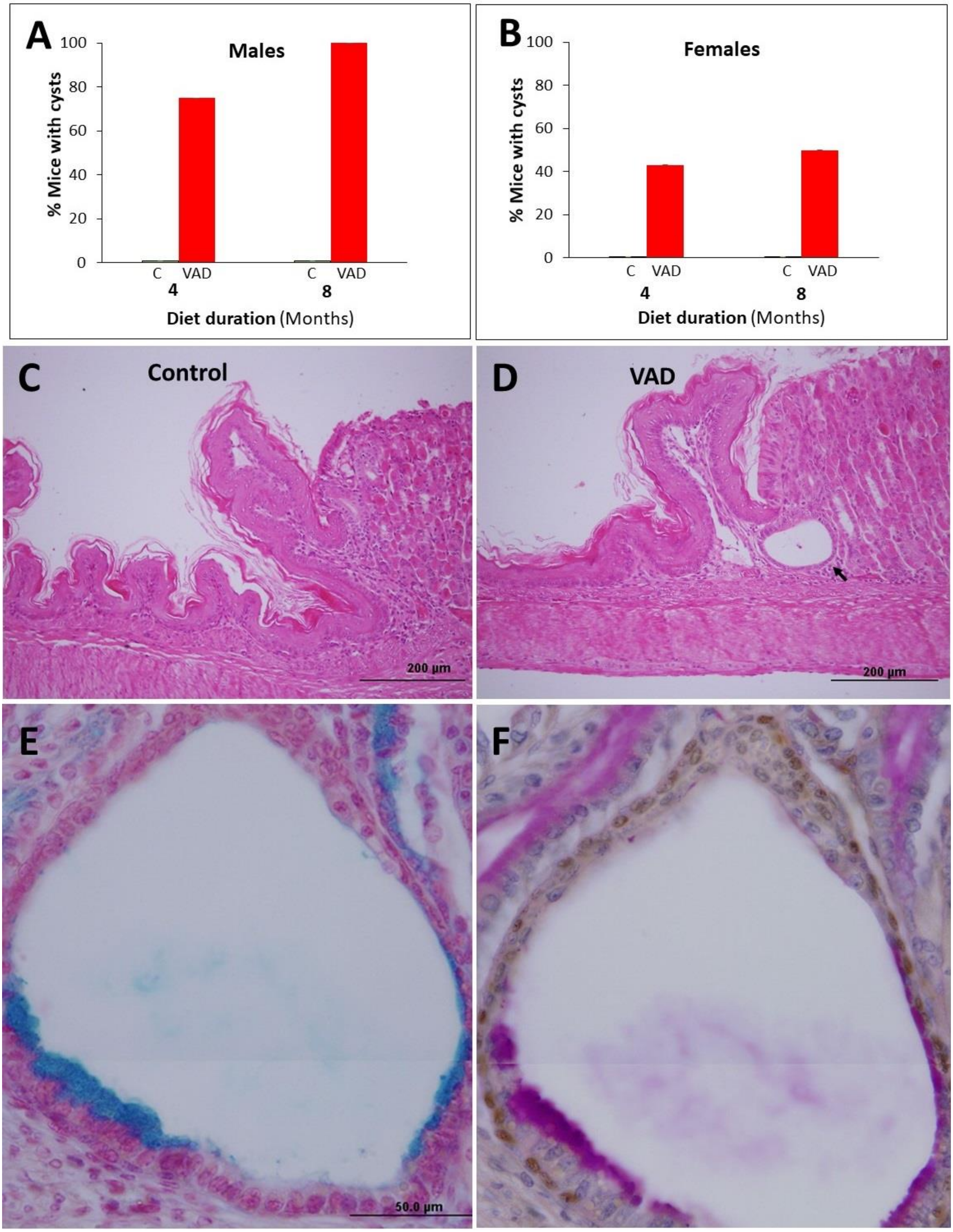
A
Males
100
80
60
40
20
0
% Mice with cysts
C VAD
4
C VAD
8
Diet duration (Months)
B
Females
100
80
60
40
20
0
% Mice with cysts
C VAD
4
C VAD
8
Diet duration (Months)
C
Control
200 µm
D
VAD
200 µm
E
50.0 µm
F

Figure S1. Percentages and histological features of cyctic dilatations. The percentage of mice with stomachs showing cystic dilations in male (A) and female (B) mice at 4 and 8 months. Tissue sections of 4-month control (C) and VAD (D) stomachs stained with hematoxylin & eosin showing the transition zone or limiting ridge between the fundus and glandular stomach with a single small cyst (arrow) in the VAD tissue. Serial sections of the 8-month VAD gastric mucosal cyct showing Alcian blue staining in luminal cells (E) and immunolocalization of p63 with positive brown nuclei in the basal layer and PAS-psitive luminal cells(F). Scale bar = 200 μm (C,D) and 50 μm (E,F)

Figure S2

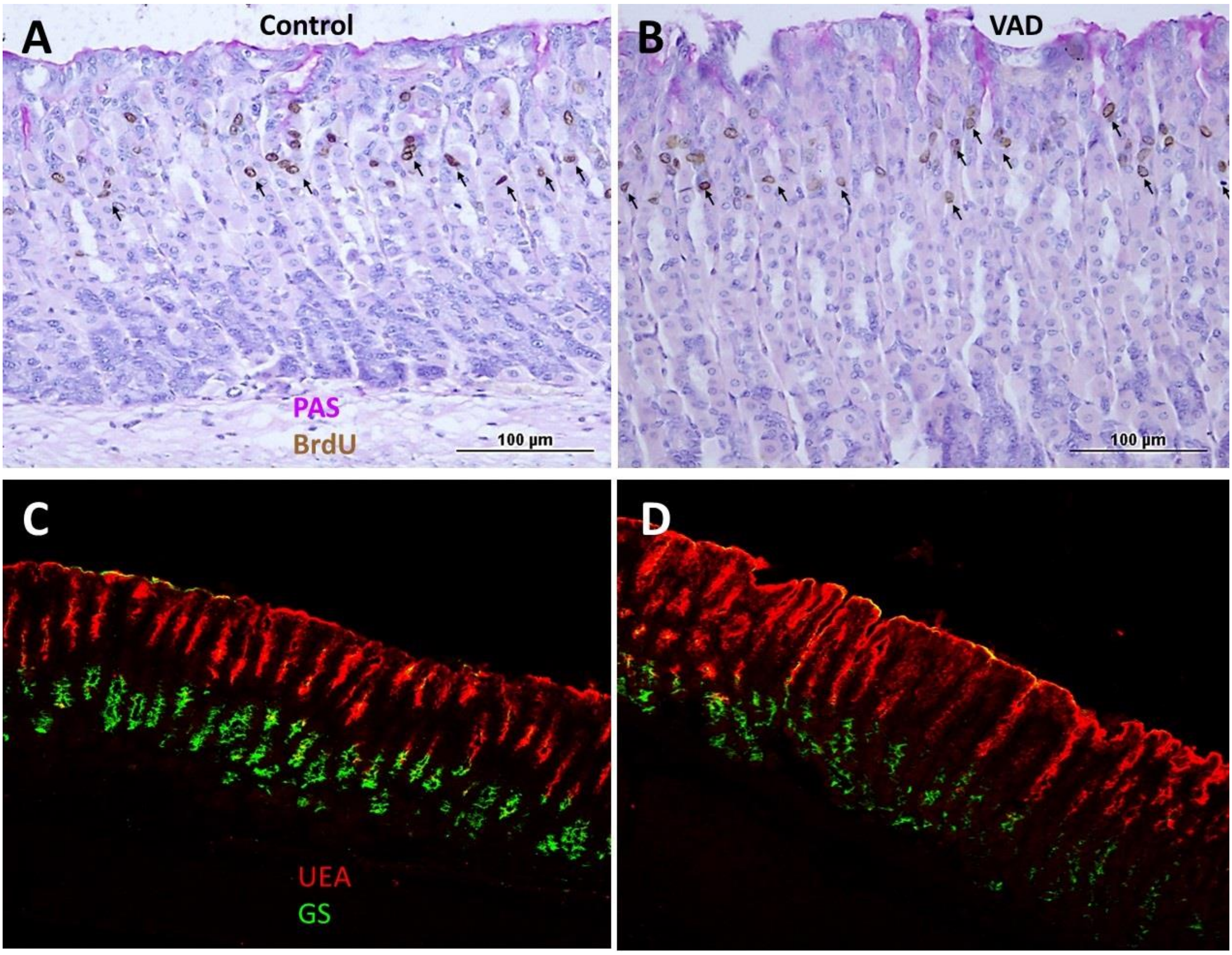

Figure S2. BrdU and lectin labeling of 4-month control and VAD mice. Gastric mucosal tissues from 4-month control (A) and VAD (B) male mice with immunohistochemical labeling showing BrdU incorporation. Tissues are counterstained with PAS. The brown colour indicates BrdU positive nuclei (arrows). Lectin labeling of mucus-secreting pit and neck cells in the gastric glands of 4-month control (C) and VAD (D) mice. Rhodamine-conjugated UEA (red) and fluorescein isothiocyanate-conjugated GS (green) lectins bind to pit and neck cells, respectively in the gastric mucosa of control (C) and VAD (D) male mice. Scale bars = 100 µm (A,B) and 200 µm (C,D)

Figure S3

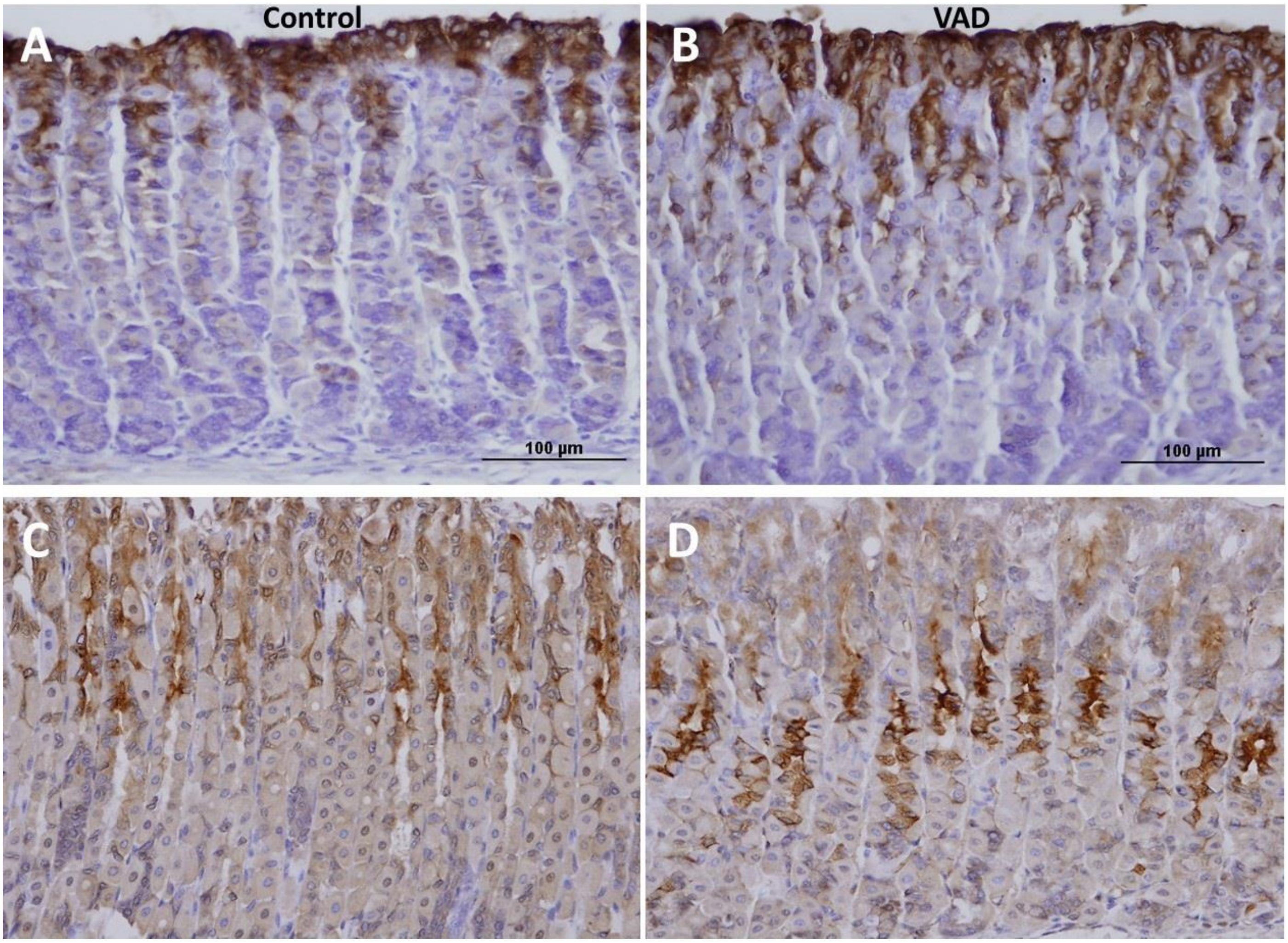

Figure S3. TFF1 and TFF2 labeling in 4-month control and VAD mice. Immunolocalization of TFF1 (A,B) and TFF2 (C,D) in the gastric glands of 4-month control (A,C) and VAD (B,D) mice. TFFs-immunolabeled cells appear brownish and cells expressing TFF1 line the luminal surface and the gastric pits and are more abundant in VAD mice (B). TFF2 is located in the cells of the neck segments of the gastric glands and demonstrates an increased labeling intensity in VAD tissues (D). Scale bars = 100 μm

Figure S4

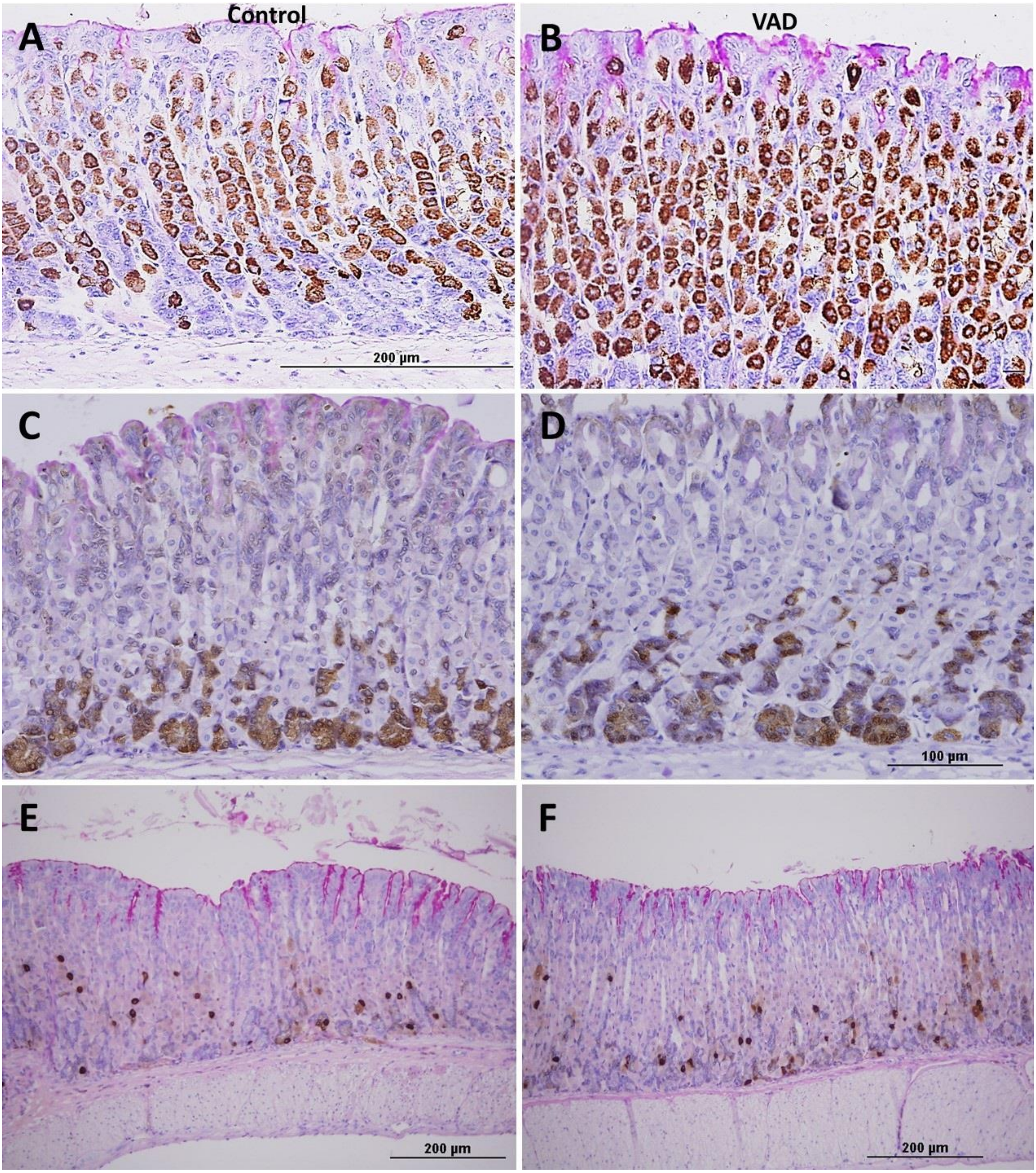

Figure S4. Parietal, zymogenic and enteroendocrine cell labeling in 4-month control and VAD mice. The gastric mucosae of 4-month control (A,C,E) and VAD (B,D,F) mice showing mmunolabeling of H^+,K^+-ATPase (A,B) in parietal cells which appear brownish and scattered along the gastric glands. Immunolocalizations of pepsinogen (C,D) and ghrelin (E,F)

respectively show basal brownish zymogenic cells and scattered enteroendocrine cells. Scale bars = 200 (A,B,E,F) and 100 (C,D) μm
